# Potential role of tirzepatide towards Covid-19 infection in diabetic patients: a perspective approach

**DOI:** 10.1007/s10787-023-01239-4

**Published:** 2023-05-19

**Authors:** Gaber El-Saber Batiha, Hayder M. Al-kuraishy, Ali I. Al-Gareeb, Nada A. Ashour, Walaa A. Negm

**Affiliations:** 1 Department of Pharmacology and Therapeutics, Faculty of Veterinary Medicine, Damanhour University, AlBeheira, P.O. Box 22511, Damanhour, Egypt; 2 Department of Clinical Pharmacology and Medicine, College of Medicine, AL-Mustansiriyia University, P.O. Box 14132, Baghdad, Iraq; 3 Department of Clinical Pharmacology and Toxicology, Faculty of Pharmacy, Tanta University, Tanta, 31527 Egypt; 4 Department of Pharmacognosy, Faculty of Pharmacy, Tanta University, Tanta, 31527, Egypt

**Keywords:** Tirazepatide, Glucose-dependent insulinotropic polypeptide, Glucagon-like peptide-1

## Abstract

In Covid-19, variations in fasting blood glucose are considered a distinct risk element for a bad prognosis and outcome in Covid-19 patients. Tirazepatide (TZT), a dual glucagon-like peptide-1 (GLP-1)and glucose-dependent insulinotropic polypeptide (GIP) receptor agonist may be effective in managing Covid-19-induced hyperglycemia in diabetic and non-diabetic patients. The beneficial effect of TZT in T2DM and obesity is related to direct activation of GIP and GLP-1 receptors with subsequent improvement of insulin sensitivity and reduction of body weight. TZT improves endothelial dysfunction (ED) and associated inflammatory changes through modulation of glucose homeostasis, insulin sensitivity, and pro-inflammatory biomarkers release. TZT, through activation of the GLP-1 receptor, may produce beneficial effects against Covid-19 severity since GLP-1 receptor agonists (GLP-1RAs) have anti-inflammatory and pulmoprotective implications in Covid-19. Therefore, GLP-1RAs could effectively treat severely affected Covid-19 diabetic and non-diabetic patients. Notably, using GLP-1RAs in T2DM patients prevents glucose variability, a common finding in Covid-19 patients. Therefore, GLP-1RAs like TZT could be a therapeutic strategy in T2DM patients with Covid-19 to prevent glucose variability-induced complications. In Covid-19, the inflammatory signaling pathways are highly activated, resulting in hyperinflammation. GLP-1RAs reduce inflammatory biomarkers like IL-6, CRP, and ferritin in Covid-19 patients. Therefore, GLP-1RAs like TZ may be effective in Covid-19 patients by reducing the inflammatory burden. The anti-obesogenic effect of TZT may reduce Covid-19 severity by ameliorating body weight and adiposity. Furthermore, Covid-19 may induce substantial alterations in gut microbiota. GLP-1RA preserves gut microbiota and prevents intestinal dysbiosis. Herein, TZT, like other GLP-1RA, may attenuate Covid-19-induced gut microbiota alterations and, by this mechanism, may mitigate intestinal inflammation and systemic complications in Covid-19 patients with either T2DM or obesity. As opposed to that, glucose-dependent insulinotropic polypeptide (GIP) was reduced in obese and T2DM patients. However, activation of GIP-1R by TZT in T2DM patients improves glucose homeostasis. Thus, TZT, through activation of both GIP and GLP-1, may reduce obesity-mediated inflammation. In Covid-19, GIP response to the meal is impaired, leading to postprandial hyperglycemia and abnormal glucose homeostasis. Therefore, using TZT in severely affected Covid-19 patients may prevent the development of glucose variability and hyperglycemia-induced oxidative stress. Moreover, exaggerated inflammatory disorders in Covid-19 due to the release of pro-inflammatory cytokines like IL-1β, IL-6, and TNF-α may lead to systemic inflammation and cytokine storm development. Besides, GIP-1 inhibits expression of IL-1β, IL-6, MCP-1, chemokines and TNF-α. Therefore, using GIP-1RA like TZT may inhibit the onset of inflammatory disorders in severely affected Covid-19 patients. In conclusion, TZT, through activation of GLP-1 and GIP receptors, may prevent SARS-CoV-2-induced hyperinflammation and glucose variability in diabetic and non-diabetic patients.

## Introduction

The most likely origin of an acute respiratory illness known as coronavirus disease in 2019 (Covid-19) is a new coronavirus known as severe acute respiratory syndrome CoV type 2 (SARS-CoV-2) (Al-kuraishy et al. 2022a, b, c; Babalghith et al. 2022a, b). SARS-CoV-2 exploits specific receptors for entry to human cells. Among the most common receptors is an angiotensin-converting enzyme type 2 (ACE2) (Al-kuraishy et al*.* 2022b; Alkhayyat et al. 2022). Cell damage and hyperinflammation are caused by a sequence of inflammatory cellular processes that follow the interaction of SARS-CoV-2 with ACE2. Numerous cellular systems, such as enterocytes, cardiomyocytes, lung alveolar cells, neurons, and testes, express and are dispersed with ACE2 (Al-kuraishy et al. 2020a, b; 2022a, b, c).

In 85% of patients, the clinical presentation of Covid-19 is predominately asymptomatic or accompanied by minor symptoms. However, due to the development of acute lung injury, 15% of Covid-19 patients presented with a moderate-severe type. (ALI). In addition, the development of acute respiratory distress syndrome (ARDS) may cause 5% of Covid-19 patients to become critically ill and require persistent breathing (Al-kuraishy et al. 2022a, b; Al-Thomali et al. 2022).

Middle East Respiratory Syndrome CoV (MERS-CoV) and SARS-CoV are substantially similar to one another and share 80% and 60% genetic similarity, respectively. SARS-CoV-2 is highly similar at the genomic level with bat CoV 96%. Nevertheless, SARS-CoV-2 is 20 times higher in use and binds ACE2 than other CoVs with succeeding down-regulation of these receptors (Babalghith et al. 2022a, b). ACE2 is a peptidase metabolizes vasoconstrictor angiotensin II (Ang II) to the vasodilator Ang1-7 and Ang1-9 (Al-kuraishy et al. 2022a). During SARS-CoV-2 infection, ACE2 is down-regulated, which causes vasoconstriction and the emergence of inflammatory, oxidative, and endothelial problems (Al-Kuraishy et al. 2022a, b, c) SARS-CoV-2-induced OS activates activation of different signaling pathways, which counterbalances this type of complication.

In Covid-19, variations in fasting blood glucose are regarded as an independent risk factor for bad prognosis and outcome in Covid-19 patients (Al-kuraishy et al. 2021). A variation in blood glucose is linked with the exaggeration of systematic inflammation, even in patients without diabetes (Al-kuraishy et al. 2021). In addition, using corticosteroids to manage severe Covid-19 is associated with hyperglycemia, which may lead to critical complications (Carranza-zavala and Manrique-franco 2020; Al-Kuraishy et al. 2022a, b). Therefore, controlling blood glucose is necessary for the management of Covid-19 patients. Using oral hypoglycemic agents and insulin to manage Covid-19-induced hyperglycemia may not associate with strict glucose control. Therefore, we hypothesized that tirazepatide dual glucagon-like peptide-1 and glucose-dependent insulinotropic polypeptide (GIP) receptor agonists might effectively manage Covid-19-induced hyperglycemia in diabetic and non-diabetic patients.

## Tirzepatide pharmacology

**Fig. 1:**
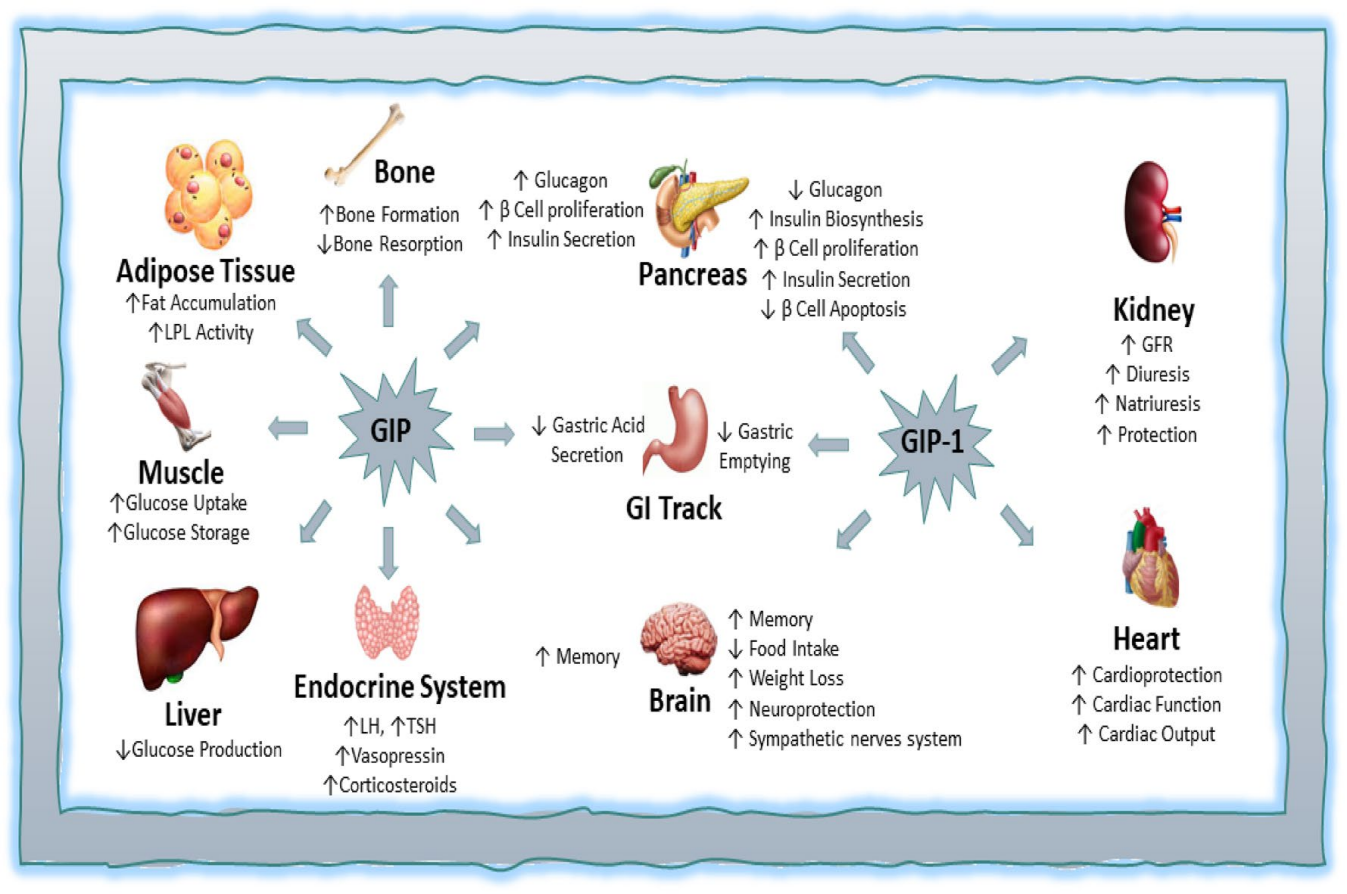
Mechanism of Tirzepatide

Tirzepatide (TZT) is a synthetic 4.8 kDa, 39 amino acids analogue of GIP that stimulates insulin release from pancreatic β cells (Urva et al. 2021). TZT is chemically modified by lipidation to increase its stability and cellular uptake. It will complete a phase III clinical trial in 2021 to manage type 2 diabetes mellitus (T2DM) (Urva et al. 2021; K.K and Kobe 2021). TZT acts by activating GIP and GLP-1 receptors, though it activated GIP more than GLP-1 (Fig. 1). It also stimulates the generation of cAMP, which regulates lipid and glycogen metabolisms (Pirro et al. 2022). Remarkably, TZT increases adiponectin levels following 26 weeks of 10 mg therapy. Of interest, treatment with TZT increases the expression of insulin-like growth factor (IGF) levels and associated binding proteins IGFBP1 and IGFBP2 (Thomas et al. 2021). Eli Lilly and Company originally applied TZT to control blood glucose in 2016; the FDA approved Lilly application in 2021 with a review voucher (Cummins and Us 2016; Sagonowsky 2021). After the completion of the successful trial on 28 April 2022, the company declared that TZT meets the required endpoints in the management of T2DM and overweight/obesity in non-diabetic subjects (Kellaher 2022; Frías et al. 2021).

A preliminary trial for the efficacy of TZT in the management of T2DM showed that this drug was less effective compared to semaglutide (an analogue of GLP-1) in the reduction of insulin resistance (IR) and glycated hemoglobin (HbA_1c_) (Frías et al. 2021). However, a recent meta-analysis illustrated that clinical use of TZT for 1 year in the management of obesity and glucose control was superior compared to semaglutide, dulaglutide, degludec, and insulin (Dutta et al. 2021). Patients with medullary thyroid cancer and type 2 multiple endocrine neoplasia syndrome should not use TZT (Syed 2022). The use of TZT is associated with developing some adverse effects, including nausea, vomiting, diarrhea, anorexia, abdominal pain, dyspepsia, and hypoglycemia (Min and Bain 2021). TZT is a novel once-weekly treatment with a dose of 5, 10, and 15 mg subcutaneously for treating T2DM and obesity. TZT is a safe agent with a low risk of hypoglycemia. A clinical trial involving 45 patients with renal impairments on TZT treatment showed no relationship between TZT pharmacokinetic and renal impairment (Urva et al. 2021). Therefore, there is no clinically relevant effect of renal impairment on TZT pharmacokinetics, and dose adjustment is not required for patients with renal impairment. The half-life of TZT is 5 days, the expected steady state is 4–5 weeks, body clearance is 0.028 L/h, and the volume of distribution is 5.27L. TZT leads to dose-dependent glucose tolerance and body weight reduction without significantly affecting the lipid profile compared with a placebo (Furihata et al. 2022).

The beneficial effect of TZT in T2DM and obesity is related to the direct activation of GIP and GLP-1 receptors with subsequent improvement of insulin sensitivity and reduction of bodyweight (Chavda et al. 2022; Min and Bain 2021). Reduction of bodyweight and improvement of insulin sensitivity may inhibit the release of pro-inflammatory biomarkers and propagation of systemic inflammatory disorders in patients with cardiovascular complications (Wilson et al. 2020).

## Tirzepatide and Covid-19

TZT improves ED and associated inflammatory changes through the modulation of glucose homeostasis, insulin sensitivity, and release of pro-inflammatory biomarkers. A clinical trial of once-weekly TZT in T2DM patients illustrated that at 26 weeks of treatment, TZT led to the significant reduction of intercellular adhesion molecule 1 (ICAM-1), growth differentiation factor 15 (GDF-15), chitosan-3 like protein 1 (YKL-40), leptin and CRP compared to the baseline values. Remarkably, CRP, YKL-40, and ICAM-1 were rapidly reduced within the first 4^th^ weeks of treatment, while leptin was gradually reduced till the time of trial. Of note, IL-6 serum level was not significantly affected by TZT treatment in this trial (Wilson et al. 2020). A study conducted by Hartman et al. to evaluate the potential effect of TZT on the biomarkers of non-alcoholic steatohepatitis in T2DM patients showed that TZT treatment increases adiponectin and reduces related inflammation in T2DM patients. Remarkably, procollagen III is keratin-18 reduced following TZT treatment in T2DM patients with non-alcoholic steatohepatitis (Hartman et al. 2020). These findings proposed the anti-inflammatory effects of TZT against the development of ED and liver inflammation.

In Covid-19, ED (a hallmark of the disease) and activation of adhesion molecules trigger the progression of immunothrombosis and the development of critical complications (Bonaventura et al. 2021; Al-kuraishy et al. 2020a, b). The increasing level of ICAM-1, GDF-15, and YKL-40 is associated with poor clinical outcomes in Covid-19 patients (Babalghith Al-kuraishy et al. 2022a, b; Ramadan et al. 2022; Sharma et al. 2020). Of note, Covid-19 leads to the induction of fibrotic remodeling secondary to lobar micro-ischemia with the development of fibrotic interstitial changes. In this state, biomarkers of lung fibrosis are increased, like procollagen III and GDF15, which reflect the underlying lung injury and associated fibrosis (Ackermann et al. 2022).

Moreover, TZT increases anti-inflammatory adiponectin and IGF (Thomas et al. 2021), which are highly deranged in Covid-19, leading to exaggerated inflammatory disorders (Al-kuraishy et al. 2020a, b; Varol et al. 2014). Therefore, TZT can reduce Covid-19 severity by modulating adiponectin and IGF.

These verdicts suggest that TZT could effectively manage Covid-19 by modulation of inflammatory pathways and regulating adiponectin and IGF.

### Tirzepatide and GLP-1 in Covid-19

Regardless of its cause, glucose variability triggers the development of oxidative stress and the release of pro-inflammatory cytokines (Nusca et al. 2018). Of interest, poor glycemic control alters the interaction between innate and adaptive immune response with the subsequent promotion of infectious/inflammatory processes and viral replication, as in SARS-CoV-2 infection (Villarreal-Calderón et al. 2019; Marfella et al. 2022). Therefore, many studies reported that T2DM patients were associated with developing serious complications during SARS-CoV-2 infection (Batista et al. 2021). Thus, good glycemic control is correlated with low mortality in T2DM Covid-19 patients compared to Covid-19 patients with poor glycemic control. A retrospective study illustrated that T2DM Covid-19 patients with blood glucose 3.9–10.0 mml/l were associated with low mortality (Zhu et al. 2020). It has been shown that Covid-19 patient mortality related to T2DM is linked with higher HbA_1c_ above 7.65% (Holman et al. 2020). Notably, strict glucose control may increase the risk of hypoglycemia and augment Covid-19 mortality in T2DM patients (Son et al. 2022). Thomas et al. observed that TZT strictly improves glucose homeostasis and prevents glucose variability via modulation of insulin sensitivity and pancreatic β cell function. A multicenter, retrospective study revealed that TZT was more effective than dulaglutide in improving glucose homeostasis in T2DM patients (Thomas et al. 2021). Therefore, TZT could effectively control blood glucose in T2DM patients with Covid-19 through modulation of insulin sensitivity and pancreatic β cell function, which are highly deranged in severe Covid-19 (Ilias et al. 2021).

On the other hand, TZT, through activation of the GLP-1 receptor, may produce beneficial effects against Covid-19 severity. Since GLP-1 receptor agonists (GLP-1RAs) have anti-inflammatory and pulmoprotective effects, they may effectively manage ALI/ARDS and hyperinflammation in Covid-19 (Belančić et al. 2021). Therefore, GLP-1RAs could effectively treat severely affected Covid-19 diabetic and non-diabetic patients. GLP-1RAs are classified as short-acting, like exenatide, and long-acting, like liraglutide. They are used subcutaneously weekly (Nauck et al. 2021). However, orally active GLP-1RA is semaglutide which was recently approved for the treatment of T2DM patients (Thethi et al. 2020). Of note, using GLP-1RAs in T2DM patients prevents glucose variability, a common finding in Covid-19 patients (Nauck et al. 2021). Thus, GLP-1RAs like TZT could be a therapeutic strategy in T2DM patients with Covid-19 to prevent glucose variability-induced complications.

Of note, GLP-1RAs reduce inflammatory biomarkers like IL-6, CRP, and ferritin in Covid-19 patients. Therefore, GLP-1RAs like TZ may be effective in Covid-19 patients by reducing the inflammatory burden (Katsiki and Ferrannini 2020). Remarkably, GLP-1Rs are highly expressed in various tissues, including pancreatic β cells, brain, endothelium, lung, GIT, kidneys, and immune cells (Baggio and Drucker 2021). Activation of GLP-1Rs on the immune cells results in reduced expression of various inflammatory signaling pathways like nod-like receptor pyrin 3 (NLRP3) inflammasome and nuclear factor kappa B (NF-κB) with subsequent inhibition release of pro-inflammatory cytokines like IL-6 and tumor necrosis factor-alpha (TNF-α) (Wan and Sun 2019; Tsukahara et al. 2015). In addition, activation of GLP-1Rs has anti-inflammatory effects via stimulation of AMPK, cAMP, endothelial nitric oxide synthase (eNOS), and suppression of chemokine expression (Jin and Liu 2020). Besides, in Covid-19, inflammatory signaling pathways like NLRP3 inflammasome and NF-κB are highly activated, leading to hyperinflammation and the development of cytokine storms (Al-kuraishy et al*.* 2022a, b). In this state, TZT may effectively reduce the risk of cytokine storm development through the activation of GLP-1RAs.

Furthermore, stimulation of GLP-1RAs by specific agonists may improve airway inflammation by inhibiting mucus production and cytokine production. Preclinical studies demonstrated that GLP-1RAs attenuate experimental ALI in mice (Zhu et al. 2015). Likewise, GLP-1RAs improve respiratory function in T2DM patients regardless of blood glucose. A prospective cohort study involving 32 T2DM patients on metformin monotherapy or metformin plus GLP-1RA illustrated that metformin plus GLP-1RA over 24 months of therapy was superior to metformin monotherapy (Rogliani et al. 2019). These findings suggest the pulmoprotective effect of GLP-1RAs. Thus, TZT use in Covid-19 patients with T2DM may reduce the risk of ALI/ARDS through modulation of airway inflammation.

Moreover, GLP-1RAs reduce bodyweight and decrease the burden of obesity on Covid-19 pathogenesis. The anti-obesogenic effect of GLP-1RAs is mediated by direct inhibition of the feeding center and indirectly through modulation of energy expenditure (Jepsen and Christensen 2021). Long-term use of GLP-1RAs can modulate obesity-mediated inflammation, immune dysfunction, and chronic low-grade inflammation in obese subjects developing Covid-19 (De Lorenzo et al. 2021). A meta-analysis involving 50 relevant published articles showed that obesity is regarded as an independent risk factor for Covid-19 severity (Aghili et al. 2021). Thus, the anti-obesogenic effect of TZT may reduce Covid-19 severity by ameliorating body weight and adiposity (Samms et al. 2021). The experimental study demonstrated that TZT effectively reduced the body weight in mice (Aghili et al. 2021; Samms et al. 2021). In this state, TZT in obese subjects with or without T2DM could be a prophylactic measure against the development of Covid-19 in obesity.

Furthermore, Covid-19 may induce substantial alterations of gut microbiota due to the direct invasion of enterocytes with the progression of intestinal inflammation. Systemic hyperinflammation, hypercytokinemia, and the development of cytokine storms may affect intestinal microbiota (Jung and Jung 2022). In addition, the development of ALI/ARDS with lung inflammation affects the intestinal integrity through the lung-gut axis (Al-Kuraishy et al. 2022a, b). Alteration of gut microbiota induces systemic inflammation with augmentation of ALI/ARDS in severely affected Covid-19 (Sencio et al. 2021). Therefore, modulation of gut microbiota by administration of probiotics and prebiotics could be a new strategy to prevent intestinal inflammation and systemic complications through modulation of inflammatory signaling pathway (Venegas-Borsellino et al. 2021). Remarkably, T2DM and obese patients have altered gut microbiota toward pathogenic species compared to the healthy controls (Verma 2022). Abnormal gut microbiota in patients with T2DM and obesity promote abnormal intestinal permeability with the development of endotoxemia and systemic complications (Venegas-Borsellino et al. 2021). Therefore, abnormal gut microbiota in patients with T2DM and obesity could be an independent risk factor for developing Covid-19 severity. In this bargain, it has been shown that GLP-1RA liraglutide positively modulates gut microbiota in patients with T2DM and obesity (Verma 2022). In addition, GLP-1RA preserves gut microbiota and prevents intestinal dysbiosis (Megur et al. 2022; Shang et al. 2021). Herein, TZT, like other GLP-1RA, may attenuate Covid-19-induced gut microbiota alterations and, by this mechanism, may mitigate intestinal inflammation and systemic complications in Covid-19 patients with either T2DM or obesity.

Moreover, the binding of SARS-CoV-2 with ACE2 is regarded as the basic point in the pathogenesis of Covid-19 (Mutter et al. 2020). It has been suggested in the early Covid-19 pandemic that overexpression may increase the risk of SARS-CoV-2 infection (Peron and Nakaya 2020). This suggestion was wrong since drugs that increase ACE2 expressions, like ibuprofen and angiotensin receptor blockers (ARBs), are protective rather than harmful when used in Covid-19 (Kelleni 2020; Poutoglidou et al. 2021). However, the potential effects of GLP-1RA on the ACE2 expression was revealed by preclinical studies that were not confirmed in human. For example, GLP-1RA liraglutide increases ACE2 expression in rats (Romaní-Pérez et al. 2015). At the same time, liraglutide induces the expression of anti-inflammatory Ang1-7 (Fandiño et al. 2018). Interestingly, ACE2 overexpression by GLP-1RA promotes the generation of anti-inflammatory Ang1-7 and reduces pro-inflammatory AngII (Romaní-Pérez et al. 2015; Fandiño et al. 2018). Therefore, GLP-1RA TZT may reduce airway inflammation and systemic disorders through the upregulation of ACE2.

TZT, through modulation of the GLP-1 effect, may reduce Covid-19 associated glucose variability, inflammatory changes, and airway inflammation.

## Tirzepatide and GIP in Covid-19

GIP is also known as a gastric inhibitory polypeptide, secreted from K cells and found in the mucosa of the jejunum and duodenum. GIP-1 is stimulated by meal intake and glucose-induced hyperosmolarity in the duodenum; it activates insulin secretion and inhibits gastric acid secretion (Killion et al. 2020). GIP acts on GIP receptors (GIPR) which are highly expressed in the pancreas, CNS, bone, and adipose tissue (Zhang et al. 2021). The effect of GIP on inflammation is controversial. For example, GIP analogue inhibits inflammatory cells and adipose tissue inflammation. The experimental study demonstrated that circulating neutrophils and pro-inflammatory monocytes are inhibited by GIP analogue in mice. Therefore, through inhibition of adipose tissue-induced inflammation, GIP can attenuate the development of insulin resistance (IR) (Varol et al. 2014). Similarly, GIP has an anti-inflammatory effect, as evidenced by the suppression of macrophage infiltration and the development of atherosclerosis in mice (Nogi et al. 2012).

In contrast, GIP augments cytokine expression in adipocytes. The circulating level of GIP is increased in obesity as it regulates lipid metabolism and adipocyte biology. GIP triggers adipocyte inflammation with the development of IR. Thus, GIP is implicated in obesity-induced IR and the development of T2DM (Nie et al. 2012). Skrha et al. (2010) found that GIP level was reduced in obese and T2DM patients. However, activation of GIPR by TZT in T2DM patients improves glucose homeostasis within 12 weeks (Frias et al. 2020). Notoriously, both GIPR agonists and antagonists lead to weight loss mainly when co-administrated with GLP-1 agonists or analogues (Killion et al. 2020). Since TZT activates both GIP and GLP-1, it induces a remarkable reduction in body weight, unlike GIPR agonist, which is implicated in the development of obesity when used alone (Seino and Yamazaki 2022). GIP promotes fatty acid synthesis and glucose uptake in adipose tissue with the development of obesity (Getty-Kaushik et al. 2006). These findings suggest that GLP modulates the effect of GIP-1 toward the obesogenic effect. Thus, TZT, through activation of both GIP and GLP-1, may reduce obesity-mediated inflammation.

In Covid-19, GIP response to the meal is impaired, leading to postprandial hyperglycemia and abnormal glucose homeostasis (Mazucanti and Egan 2020). This effect might be due to direct enterocyte injury by SARS-CoV-2 or indirectly due to hyperinflammation and cytokine storm (Jung and Jung 2022). Therefore, using TZT in severely affected Covid-19 patients may prevent the development of glucose variability and hyperglycemia-induced oxidative stress.

In addition, in many preclinical studies, GIP promotes cortisol production and release through induction of adrenal cortex steroidogenesis (Lecoq et al. 2018; Swords et al. 2005). GIP mediates the metabolic effect of corticosterone (Bates et al. 2012). Therefore, deficiency of GIP attenuates the development of ovariectomized-induced obesity in mice (Isken et al. 2008). This effect could be useful in maintaining cortisol levels, which is reduced in Covid-19 patients. However, cortisol level is not correlated with the outcomes of Covid-19 patients with ARDS (Marpaung et al. 2022). A prospective study conducted by Güven and Gültekin (2021) showed that high cortisol level in hospitalized Covid-19 patients was associated with high mortality. Notably, a prospective study confirmed that the hypothalamic–pituitary–adrenal axis is impaired in hospitalized Covid-19 patients, as reflected by low ACTH and cortisol levels (Alzahrani et al. 2021). These findings suggest potential hypothalamic–pituitary–adrenal axis abnormality due to SARS-CoV-2-induced central adrenal insufficiency. A double-blind crossover clinical trial illustrated that GLP-1RA did not affect the hypothalamic–pituitary–adrenal axis following long-term use in normal healthy volunteers (Winzeler et al. 2020). Therefore, the effect of TZT on cortisol levels is mainly mediated by GIPR activation. Thus, normalization of cortisol levels by TZT through activation of GIPR may prevent variations in cortisol levels in severely affected Covid-19 patients.

Moreover, exaggerated inflammatory disorders in Covid-19 due to the release of pro-inflammatory cytokines like IL-1β, IL-6, and TNF-α may lead to the development of systemic inflammation and cytokine storm (Zanza et al. 2022). Besides, different preclinical studies confirmed that GIP inhibits the expression of IL-1β, IL-6, MCP-1, chemokines, and TNF-α (Varol et al. 2014). Therefore, using GIP-1RA like TZT may reduce the development of inflammatory disorders in severely affected Covid-19 patients.

TZT, through activation of GLP-1 and GIP receptors, may prevent SARS-CoV-2-induced hyperinflammation and glucose variability in diabetic and non-diabetic patients. Herein, we are exciting researchers to do a clinical trial to elucidate the possible beneficial effect of TZT in managing Covid-19, mainly in patients with T2DM and obesity.

The present hypothesis has many limitations, including no retrospective, prospective and clinical trial studies evaluating the effect of TZT in the management of Covid-19. In addition, this new drug needs long-term follow-up and post-marketing surveillance to detect remote unexpected adverse effects.

## Conclusions

SARS-CoV-2 uses the ACE2 vulnerability to get into human cells. Cell damage and hyperinflammation are caused by a sequence of inflammatory cellular processes that follow the interaction of SARS-CoV-2 with ACE2. In Covid-19, variations in fasting blood glucose are considered an independent risk factor for poor prognosis and outcome in Covid-19 patients. Therefore, controlling blood glucose is necessary in managing Covid-19 patients. Tirazepatide (TZT), a dual glucagon like peptide-1 (GLP-1)and glucose-dependent insulinotropic polypeptide (GIP) receptor agonist, may be effective in the management of Covid-19-induced hyperglycemia in diabetic and non-diabetic patients. The beneficial effect of TZT in T2DM and obesity is related to the direct activation of GIP and GLP-1 receptors with subsequent improvement of insulin sensitivity and reduction of bodyweight. TZT improves endothelial dysfunction (ED) and associated inflammatory changes through modulation of glucose homeostasis, insulin sensitivity, and release of pro-inflammatory biomarkers. Therefore, TZT could effectively control blood glucose in T2DM patients with Covid-19 through modulation of insulin sensitivity and pancreatic β cell function, which are highly deranged in severe Covid-19. TZT, through activation of the GLP-1 receptor, may produce beneficial effects against Covid-19 severity. Since GLP-1 receptor agonists (GLP-1RAs) have anti-inflammatory and pulmoprotective effects, they may effectively manage ALI/ARDS and hyperinflammation in Covid-19. Therefore, GLP-1RAs could effectively treat severely affected Covid-19 diabetic and non-diabetic patients. Of note, using GLP-1RAs in T2DM patients prevents glucose variability, a common finding in Covid-19 patients. Thus, GLP-1RAs like TZT could be a therapeutic strategy in T2DM patients with Covid-19 to prevent glucose variability-induced complications.

In Covid-19, inflammatory signaling pathways like NLRP3 inflammasome and NF-κB are highly activated, leading to hyperinflammation and the development of cytokine storms. GLP-1RAs reduce inflammatory biomarkers like IL-6, CRP, and ferritin in Covid-19 patients. Therefore, GLP-1RAs like TZ may be effective in Covid-19 patients by reducing the inflammatory burden. In this state, TZT may effectively reduce the risk of cytokine storm development through the activation of GLP-1RAs. Thus, TZT use in Covid-19 patients with T2DM may reduce the risk of ALI/ARDS through modulation of airway inflammation.

Moreover, GLP-1RAs reduce bodyweight and decrease the burden of obesity on Covid-19 pathogenesis. Therefore, the anti-obesogenic effect of TZT may reduce Covid-19 severity by ameliorating body weight and adiposity. In this state, TZT use in obese subjects with or without T2DM could be a prophylactic measure against the development of Covid-19 in obesity. Furthermore, Covid-19 may induce substantial alterations of gut microbiota due to the direct invasion of enterocytes with the progression of intestinal inflammation. Abnormal gut microbiota in patients with T2DM and obesity promote abnormal intestinal permeability with the development of endotoxemia and systemic complications. Therefore, abnormal gut microbiota in patients with T2DM and obesity could be an independent risk factor for developing Covid-19 severity. Herein, TZT, like other GLP-1RA, may attenuate Covid-19-induced gut microbiota alterations, and this mechanism may mitigate intestinal inflammation and systemic complications in Covid-19 patients with either T2DM or obesity.

Moreover, the binding of SARS-CoV-2 with ACE2 is regarded as the basic point in the pathogenesis of Covid-19. However, the potential effects of GLP-1RA on ACE2 expression were revealed by preclinical studies. Interestingly, ACE2 overexpression by GLP-1RA promotes the generation of anti-inflammatory Ang1-7 and reduces pro-inflammatory AngII. Therefore, GLP-1RA TZT may reduce airway inflammation and systemic disorders through the upregulation of ACE2.

On the other hand, GIP acts on GIP receptors (GIPR) which are highly expressed in the pancreas, CNS, bone, and adipose tissue. GIP-1 has an anti-inflammatory effect, though GIP-1 level was reduced in obese and T2DM patients. Activation of GIP-1R by TZT in T2DM patients improves glucose homeostasis. Notoriously, both GIP-R agonists and antagonists lead to weight loss mainly when co-administrated with GLP-1 agonists or analogues. Since TZT activates both GIP and GLP-1, it induces remarkable body weight reduction, unlike GIPR agonists, which are implicated in the development of obesity when used alone. These findings suggest that GLP-1 modulates the effect of GIP toward the obesogenic effect. Thus, TZT, through activation of both GIP and GLP-1, may reduce obesity-mediated inflammation. In Covid-19, GIP response to the meal is impaired, leading to postprandial hyperglycemia and abnormal glucose homeostasis. Therefore, using TZT in severely affected Covid-19 patients may prevent the development of glucose variability and hyperglycemia-induced oxidative stress.

In addition, GIP promotes cortisol production and release through the induction of adrenal cortex steroidogenesis. This effect could be useful in reducing cortisol levels in Covid-19 patients. Notably, the hypothalamic–pituitary–adrenal axis is impaired in Covid-19 patients, as reflected by low ACTH and cortisol levels. The effect of TZT on cortisol levels is mainly mediated by GIPR activation. Thus, normalization of cortisol levels by TZT through activation of GIP-1R may prevent variations in cortisol levels in severely affected Covid-19 patients.

Moreover, exaggerated inflammatory disorders in Covid-19 due to the release of pro-inflammatory cytokines like IL-1β, IL-6, and TNF-α may lead to systemic inflammation and cytokine storm development. Besides, GIP-1 inhibits expression of IL-1β, IL-6, MCP-1, chemokines and TNF-α. Therefore, using GIP-1RA like TZT may reduce the development of inflammatory disorders in severely affected Covid-19 patients.

**Fig. 2:**
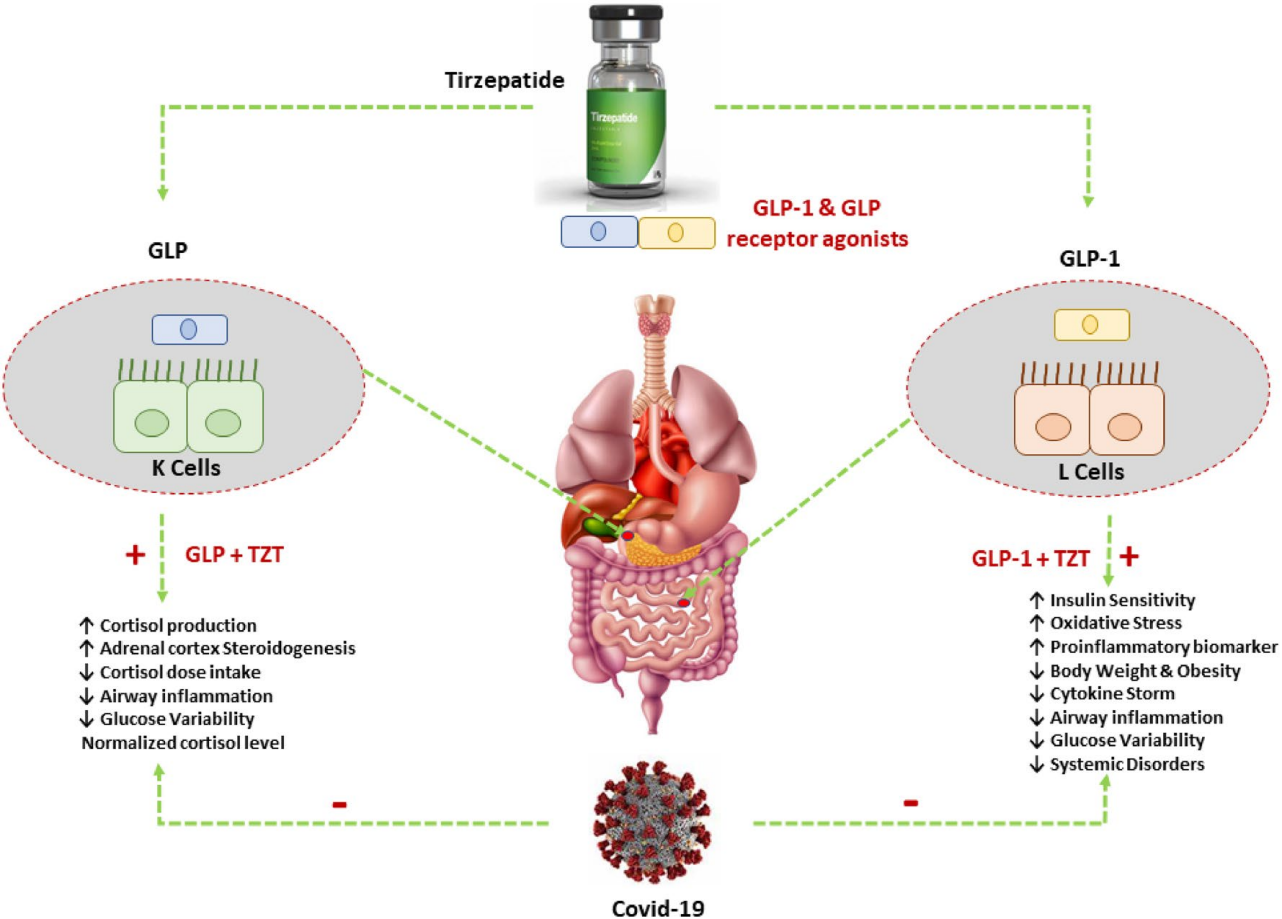
Possible mechanism of Tirzepatide in Covid-19

TZT, through activation of GLP-1 and GIP receptors, may prevent SARS-CoV-2-induced hyperinflammation and glucose variability in diabetic and non-diabetic patients (Fig. 2). Herein, we are exciting researchers to do a clinical trial to elucidate the possible beneficial effect of TZT in managing Covid-19, mainly in patients with T2DM and obesity.

## Data Availability

All data are available in the manuscript.

## References

[CR1] Ackermann M (2022). The fatal trajectory of pulmonary COVID-19 is driven by lobular ischemia and fibrotic remodelling. eBioMedicine.

[CR2] Aghili SMM (2021). Obesity in COVID-19 era, implications for mechanisms, comorbidities, and prognosis: a review and meta-analysis. Int J Obes.

[CR3] Al-kuraishy HM (2022). The potential molecular implications of adiponectin in the evolution of SARS-CoV-2: inbuilt tendency. J King Saud Univ Sci.

[CR4] Al-kuraishy HM, Al-gareeb AI, Al-hussaniy HA (2020). Neutrophil Extracellular Traps (NETs) and Covid-19: A new frontiers for therapeutic modality. Int Immunopharmacol.

[CR5] Al-kuraishy HM (2021). COVID-19 in relation to hyperglycemia and diabetes mellitus. Front Cardiovasc Med.

[CR6] Al-kuraishy HM, Al-Buhadily AK, Al-Gareeb AI (2022). Citicoline and COVID-19: vis-à-vis conjectured. Naunyn-Schmiedeberg’s Archiv Pharmacol.

[CR7] Al-kuraishy HM, Al-Gareeb AI, Elekhnawy E (2022). Dipyridamole and adenosinergic pathway in Covid-19: a juice or holy grail. Egypt J Med Human Genet.

[CR12] Al-Kuraishy HM, Al-Gareeb AI, Alexiou A (2022). 5-HT/CGRP pathway and Sumatriptan role in Covid-19. Biotechnol Genet Eng Rev.

[CR13] Al-Kuraishy HM, Al-Gareeb AI, Al-Niemi MS (2022). Calprotectin: The Link Between Acute Lung Injury and Gastrointestinal Injury in Covid-19: Ban or Boon. Curr Protein Peptide Sci.

[CR14] Al-Kuraishy HM, Al-Gareeb AI, Mostafa-Hedeab G (2022). COVID-19 and diabetes: will novel drugs for diabetes help in COVID-19?. Curr Mol Pharmacol.

[CR15] Al-Thomali AW (2022). Role of Neuropilin 1 in COVID-19 Patients with Acute Ischemic Stroke. Biomedicines.

[CR16] Alkhayyat SS (2022). Fenofibrate for COVID-19 and related complications as an approach to improve treatment outcomes: the missed key for Holy Grail. Inflamm Res.

[CR17] Alzahrani AS (2021). The Impact of COVID-19 Viral Infection on the Hypothalamic-Pituitary-Adrenal Axis. Endocrine Practice.

[CR18] Babalghith AO, Al-kuraishy HM, Al-Gareeb AI, De Waard M, Sabatier JM (2022). ‘The Potential Role of Growth Differentiation Factor 15 in COVID-19: A Corollary Subjective Effect or Not?. Diagnostics.

[CR19] Babalghith AO, Al-kuraishy HM, Al-Gareeb AI, De Waard M, Al-Hamash SM (2022). The role of berberine in Covid-19: potential adjunct therapy. Inflammopharmacology.

[CR20] Baggio LL, Drucker DJ (2021). Glucagon-like peptide-1 receptor co-agonists for treating metabolic disease. Mol Metab.

[CR21] Bates HE (2012). Gipr is essential for adrenocortical steroidogenesis; however, corticosterone deficiency does not mediate the favorable metabolic phenotype of Gipr -/- mice. Diabetes.

[CR22] Batista DV (2021). COVID-19-associated euglycemic diabetic ketoacidosis in a patient with type 2 diabetes on SGLT2 inhibitor: a case report. Diabetol Int.

[CR23] Belančić A, Kresović A, Troskot Dijan M (2021). Glucagon-like peptide-1 receptor agonists in the era of COVID -19: Friend or foe?. Clin Obes.

[CR24] Bonaventura A (2021). ‘Endothelial dysfunction and immunothrombosis as key pathogenic mechanisms in COVID-19. Nat Rev Immunol.

[CR25] Carranza-zavala B, Manrique-franco K (2020). Daily glucose variation influenced by the use of corticosteroids in COVID-19 patients treated in Lima-Peru. Diabetes Metab Syndr Clin Res Rev.

[CR26] Chavda VP, Ajabiya J, Teli D, Bojarska J, Apostolopoulos V (2022). Tirzepatide, a New Era of Dual-Targeted Treatment for Diabetes and Obesity: A Mini-Review. Molecules.

[CR27] Cummins RC, Us IN (2016) United States Patent 2(12). 10.2174/1381612043382774.(60)

[CR47] De Lorenzo A (2021). Obesity-related inflammation and endothelial dysfunction in covid-19: Impact on disease severity. J Inflamm Res.

[CR28] Dutta D (2021). Efficacy and safety of novel twincretin tirzepatide a dual GIP and GLP-1 receptor agonist in the management of type-2 diabetes: A Cochrane meta-analysis. Indian J Endocrinol Metab.

[CR29] Fandiño J (2018). Liraglutide enhances the activity of the Ace-2/Ang(1–7)/Mas receptor pathway in lungs of Male pups from food-restricted mothers and prevents the reduction of SP-A. Int J Endocrinol.

[CR30] Frias JP (2020). Efficacy and tolerability of tirzepatide, a dual glucose-dependent insulinotropic peptide and glucagon-like peptide-1 receptor agonist in patients with type 2 diabetes: A 12-week, randomized, double-blind, placebo-controlled study to evaluate different dose-escalation regimens. Diabetes Obes Metab.

[CR31] Frías JP, Landó LF, Brown K (2021). Tirzepatide versus semaglutide once weekly in patients with type 2 diabetes. New England J Med.

[CR32] Furihata K (2022). A phase 1 multiple-ascending dose study of tirzepatide in Japanese participants with type 2 diabetes. Diabetes Obes Metab.

[CR33] Getty-Kaushik L (2006). Glucose-dependent insulinotropic polypeptide modulates adipocyte lipolysis and reesterification. Obesity.

[CR34] Güven M, Gültekin H (2021). Could serum total cortisol level at admission predict mortality due to coronavirus disease 2019 in the intensive care unit? A prospective study. Sao Paulo Med J.

[CR35] Hartman ML (2020). Effects of novel dual GIP and GLP-1 receptor agonist tirzepatide on biomarkers of nonalcoholic steatohepatitis in patients with type 2 diabetes. Diabetes Care.

[CR62] Holman N, Knighton P, Kar P, O'Keefe J, Curley M, Weaver A, Barron E, Bakhai C, Khunti K, Wareham NJ, Sattar N (2020). Risk factors for COVID-19-related mortality in ¯people with type 1 and type 2 diabetes in England: a population-based cohort study. Lancet Diabetes Endocrinol.

[CR36] Ilias I (2021). Glycemia, beta-cell function and sensitivity to insulin in mildly to critically ill covid-19 patients. Medicina (Lithuania).

[CR37] Isken F (2008). Deficiency of glucose-dependent insulinotropic polypeptide receptor prevents ovariectomy-induced obesity in mice. Am J Physiol Endocrinol Metab.

[CR38] Jepsen MM, Christensen MB (2021). Emerging glucagon-like peptide 1 receptor agonists for the treatment of obesity. Expert Opin Emerging Drugs.

[CR39] Jin T, Liu M (2020). Letter to the editor: Comment on GLP-1-based drugs and COVID-19 treatment. Acta Pharmaceutica Sinica B.

[CR40] Jung HN, Jung CH (2022). The Upcoming Weekly Tides (Semaglutide vs. Tirzepatide) against Obesity: STEP or SURPASS?. J Obes Metabolic Syndrome.

[CR41] KK ELJ, Kobe HJ (2021) I8F-JE-GPGO Statistical Analysis Plan Addendum Version 2 1. Statistical Analysis Plan Addendum : I8F-JE-GPGO : A Phase 3 Study of Tirzepatide Monotherapy Compared to Dulaglutide 0 . 75 mg in Patients with Type 2 Diabetes Mellitus ( SURPASS J-mono ) Meal’, pp. 0–12.

[CR42] Katsiki N, Ferrannini E (2020). Anti-inflammatory properties of antidiabetic drugs: a “promised land” in the COVID-19 era?. J Diabetes Complications.

[CR43] Kellaher C (2022) Eli Lilly’s Tirzepatide Meets Main Endpoints in Phase 3 Obesity Study, MarketWatch. Dow Jones Newswires [Preprint]

[CR44] Kelleni MT (2020). ACEIs, ARBs, ibuprofen originally linked to COVID-19: the other side of the mirror. Inflammopharmacology.

[CR45] Killion EA (2020). Glucose-Dependent Insulinotropic Polypeptide Receptor Therapies for the Treatment of Obesity, Do Agonists = Antagonists?. Endocrine Rev.

[CR46] Lecoq A-L (2018). Adrenal GIPR expression and chromosome 19q13 microduplications in GIP-dependent Cushing’s syndrome. Annales d’Endocrinologie.

[CR48] Marfella R (2022). Does poor glycaemic control affect the immunogenicity of the COVID-19 vaccination in patients with type 2 diabetes: The CAVEAT study. Diabetes Obes Metab.

[CR49] Marpaung FR (2022). Analysis of Adrenocorticotropic Hormone and Cortisol Levels in Acute Respiratory Distress Syndrome COVID-19 Patients. Disease Markers.

[CR50] Mazucanti CH, Egan JM (2020). SARS-CoV-2 disease severity and diabetes: why the connection and what is to be done?. Immunity Ageing.

[CR51] Megur A (2022). Prebiotics as a Tool for the Prevention and Treatment of Obesity and Diabetes: Classification and Ability to Modulate the Gut Microbiota. Int J Mol Sci.

[CR52] Min T, Bain SC (2021). The Role of Tirzepatide, Dual GIP and GLP-1 Receptor Agonist, in the Management of Type 2 Diabetes: The SURPASS Clinical Trials. Diabetes Ther.

[CR53] Mutter H (2020). Renin—angiotensin system and fibrinolytic pathway in COVID—19: one—way skepticism. Biomed Biotechnol Res J.

[CR54] Nauck MA (2021). GLP-1 receptor agonists in the treatment of type 2 diabetes – state-of-the-art. Mol Metab.

[CR55] Nie Y (2012). Glucose-dependent insulinotropic peptide impairs insulin signaling via inducing adipocyte inflammation in glucose-dependent insulinotropic peptide receptor-overexpressing adipocytes. FASEB J.

[CR56] Nogi Y (2012). Glucose-dependent insulinotropic polypeptide prevents the progression of macrophage-driven atherosclerosis in diabetic apolipoprotein E-null mice. PLoS ONE.

[CR57] Nusca A (2018). Glycemic variability in the development of cardiovascular complications in diabetes. Diabetes/Metab Res Rev.

[CR58] Peron JPS, Nakaya H (2020). Susceptibility of the elderly to SARS-COV-2 infection: ACE-2 overexpression, shedding, and antibody-dependent enhancement (ADE). Clinics.

[CR59] Pirro V (2022). Effects of Tirzepatide, a Dual GIP and GLP-1 RA, on Lipid and Metabolite Profiles in Subjects with Type 2 Diabetes. J Clin Endocrinol Metab.

[CR60] Poutoglidou F, Saitis A, Kouvelas D (2021). Ibuprofen and COVID-19 disease: separating the myths from facts. Expert Rev Respirat Med.

[CR61] Ramadan AE (2022). Serum Levels of Intercellular Adhesion Molecule-1 and TNF-α in Patients with COVID-19 and Its Relation to Disease Severity. Egypt J Hospital Med.

[CR63] Rogliani P (2019). Long-term observational study on the impact of GLP-1R agonists on lung function in diabetic patients. Respirat Med.

[CR64] Romaní-Pérez M (2015). Activation of the GLP-1 receptor by liraglutide increases ACE2 expression, reversing right ventricle hypertrophy, and improving the production of SP-A and SP-B in the lungs of type 1 diabetes rats. Endocrinology (United States).

[CR65] Sagonowsky E (2021) As Lilly gears up for key 2022 launches, Trulicity, Taltz and more drive solid growth. Fierce Pharma [Preprint]

[CR66] Samms RJ (2021). GIPR agonism mediates weight-independent insulin sensitization by tirzepatide in obese mice. J Clin Investig.

[CR67] Seino Y, Yamazaki Y (2022). Roles of glucose-dependent insulinotropic polypeptide in diet-induced obesity. J Diabetes Investig.

[CR68] Sencio V, Machado MG, Trottein F (2021). The lung–gut axis during viral respiratory infections: the impact of gut dysbiosis on secondary disease outcomes. Mucosal Immunol.

[CR69] Shang J (2021). Liraglutide-induced structural modulation of the gut microbiota in patients with type 2 diabetes mellitus. PeerJ.

[CR70] Sharma N, Modak C, Singh PK, Kumar R, Khatri D, Singh SB (2020). Underscoring the immense potential of chitosan in fighting a wide spectrum of viruses: A plausible molecule against SARS-CoV-2?. Int J Biol Macromol.

[CR71] Skrha J (2010). Meal test for Glucose-dependent insulinotropic peptide (GIP) in obese and Type 2 diabetic patients. Physiol Res.

[CR72] Son KH (2022). ‘Hyperglycemia and Hypoglycemia Are Associated with In-Hospital Mortality among Patients with Coronavirus Disease 2019 Supported with Extracorporeal Membrane Oxygenation. J Clin Med.

[CR73] Swords FM (2005). The aberrant expression of the gastric inhibitory polypeptide (GIP) receptor in adrenal hyperplasia: Does chronic adrenocorticotropin exposure stimulate up-regulation of GIP receptors in Cushing’s disease?. J Clin Endocrinol Metab.

[CR74] Syed YY (2022) Tirzepatide: first approval. 10.1007/s40265-022-01746-8

[CR75] Thethi TK, Pratley R, Meier JJ (2020). Efficacy, safety and cardiovascular outcomes of once-daily oral semaglutide in patients with type 2 diabetes: The PIONEER programme. Diabetes, Obes Metab.

[CR76] Thomas MK (2021). Dual GIP and GLP-1 Receptor Agonist Tirzepatide Improves Beta-cell Function and Insulin Sensitivity in Type 2 Diabetes. J Clin Endocrinol Metab.

[CR77] Tsukahara T (2015). Tumor necrosis factor α decreases glucagon-like peptide-2 expression by up-regulating G-protein-coupled receptor 120 in crohn disease. Am J Pathol.

[CR78] Urva S (2021). Effects of Renal Impairment on the Pharmacokinetics of the Dual GIP and GLP-1 Receptor Agonist Tirzepatide. Clin Pharmacokinet.

[CR79] Varol C (2014). Long-Acting Glucose-Dependent Insulinotropic Polypeptide Ameliorates Obesity-Induced Adipose Tissue Inflammation. J Immunol.

[CR80] Venegas-Borsellino C (2021). Impact of COVID-19 on the Intestinal Microbiome. Curr Nutr Rep.

[CR81] Verma S (2022) Probiotics can be a tool to fight COVID-19. Understanding Covid-19 [Preprint]

[CR82] Villarreal-Calderón JR (2019). Interplay between the adaptive immune system and insulin resistance in weight loss induced by bariatric surgery. Oxidat Med Cell Longev.

[CR83] Wan S, Sun H (2019). Glucagon-like peptide-1 modulates RAW264.7 macrophage polarization by interfering with the JNK/STAT3 signaling pathway. Exp Ther Med.

[CR84] Wilson JM (2020). The dual glucose-dependent insulinotropic peptide and glucagon-like peptide-1 receptor agonist, tirzepatide, improves lipoprotein biomarkers associated with insulin resistance and cardiovascular risk in patients with type 2 diabetes. Diabetes Obes Metab.

[CR85] Winzeler B (2020). Effects of glucagon-like peptide-1 receptor agonists on fluid intake in healthy volunteers. Endocrine.

[CR86] Zanza C (2022). Cytokine Storm in COVID-19: Immunopathogenesis and Therapy. Medicina (Lithuania).

[CR87] Zhang Q (2021). The glucose-dependent insulinotropic polypeptide (GIP) regulates body weight and food intake via CNS-GIPR signaling. Cell Metab.

[CR88] Zhu T (2015). Glucagon like peptide-1 (GLP-1) modulates OVA-induced airway inflammation and mucus secretion involving a protein kinase A (PKA)-dependent nuclear factor-κB (NF-κB) signaling pathway in mice. Int J Mol Sci.

[CR89] Zhu L (2020). Association of Blood Glucose Control and Outcomes.pdf. Cell Metab.

